# Using Physiological Markers to Assess Comfort during Neuromuscular Electrical Stimulation Induced Muscle Contraction in a Virtually Guided Environment: Pilot Study for a Path toward Combating ICU-Acquired Weakness

**DOI:** 10.3390/s24113599

**Published:** 2024-06-03

**Authors:** Ahmad Abou-Hamde, Lauren Philippi, Eric Jones, Christian Martin, Kingsley Wu, Michael Kundell, Sunita Mathur, Alireza Sadeghian, Maryam Davoudpour, Jane Batt, Adriana Ieraci, Sharon Gabison

**Affiliations:** 1 Department of Physical Therapy, Faculty of Medicine, University of Toronto, Toronto, ON M5G 1V7, Canadalauren.philippi15@gmail.com (L.P.); ericjones_6@hotmail.com (E.J.); christianm.smcs@gmail.com (C.M.); thekingsleywu@gmail.com (K.W.); mj.kundell21@hotmail.com (M.K.); 2 School of Rehabilitation Therapy, Queen’s University, Kingston, ON K7L 3N6, Canada; sunita.mathur@queensu.ca; 3 Department of Computer Science, Toronto Metropolitan University, Toronto, ON M5B 2K3, Canada; asadeghi@ryerson.ca (A.S.); adriana.ieraci@gmail.com (A.I.); 4 Faculty of Applied Sciences and Technology, Humber College, Toronto, ON M9W 5L7, Canada; maryam.davoudpour@humber.ca; 5 Division of Respirology, Department of Medicine, Unity Health Toronto, Toronto, ON M5B 1W8, Canada; jane.batt@utoronto.ca

**Keywords:** electric stimulation, heart rate, feasibility studies, intensive care units

## Abstract

We assessed the feasibility of implementing a virtually guided Neuromuscular Electrical Stimulation (NMES) protocol over the tibialis anterior (TA) muscle while collecting heart rate (HR), Numeric Pain Rating Scale (NPRS), and quality of contraction (QoC) data. We investigated if HR, NPRS, and QoC differ ON and OFF the TA motor point and explored potential relationships between heart rate variability (HRV) and the NPRS. Twelve healthy adults participated in this cross-sectional study. Three NMES trials were delivered ON and OFF the TA motor point. HR, QoC, and NPRS data were collected. There was no significant difference in HRV ON and OFF the motor point (*p* > 0.05). The NPRS was significantly greater OFF the motor point (*p* < 0.05). The QoC was significantly different between motor point configurations (*p* < 0.05). There was no correlation between the NPRS and HRV (*p* > 0.05, r = −0.129). We recommend non-electrical methods of measuring muscle activity for future studies. The NPRS and QoC can be administered virtually. Time-domain HRV measures could increase the validity of the protocol. The variables should be explored further virtually to enhance the protocol before eventual ICU studies.

## 1. Introduction

Approximately 40% of critically ill patients develop Intensive-Care-Unit-Acquired Weakness (ICUAW) which is characterized by the rapid loss of muscle mass, impaired contractility, and the loss of peripheral nerve function [1]. ICUAW is caused by a combination of factors including prolonged immobilization, inflammation due to critical illness, and some pharmacotherapies required to sustain life such as corticosteroids [2,3]. Atrophy of lower-limb skeletal muscle can occur at a rate of 1.2–3.0% per day [4]. Persistent atrophy and impaired contractility contribute to permanent weaknesses in critical illness survivors and cause long-lasting physical functional impairments, a reduction in quality of life, and significant increases in health resource utilization [5]. 

Neuromuscular Electrical Stimulation (NMES) can preserve the strength and physical function of patients in the intensive care unit (ICU) [6] and can also be used post-ICU in rehabilitation programs. NMES treatment applies electrical pulses to the target skeletal muscles via electrodes placed on the skin [6,7,8]. Comfortable and high-quality muscle contraction produced by NMES requires the electrodes to be placed on the electrophysiological motor point of the muscle [9]. Conversely, when stimulation is delivered off the muscle motor point, the quality of contraction may decrease and cause pain [10]. Thus, NMES therapy requires application by a trained individual (e.g., physiotherapist) as well as verbal feedback from the patient confirming comfortable delivery, making it a resource-intensive treatment option and challenging in a non-responsive patient. 

In the ICU, where verbal communication with the patient can be limited due to sedation and mechanical ventilation, physiological markers such as changes in heart rate variability (HRV) can be used to provide information regarding patient comfort [11,12,13,14,15] during NMES therapy. Frequency-domain measures extracted from the heart rate (HR) signal have been shown to provide information about the balance between the sympathetic and parasympathetic division of the autonomic nervous system [12] and are shown to correlate well with the patient-reported Numeric Pain Rating Scale (NPRS), the gold standard of metrics used to quantify pain [13]. 

Cyclical lockdowns instituted in response to the COVID-19 pandemic forced the cancellation of all non-essential out-patient-based physical therapy treatment and rehabilitation programs. The COVID-19 response measures also caused the stoppage of much clinical research in academic hospitals, highlighting the need to develop physical therapy approaches and research methods adaptable to a virtual environment [16]. No studies have been conducted evaluating the use of physiologic metrics, such as HRV, to quantify responses to NMES in a remote capacity. The ability to capture and standardize physiologic metrics from NMES administered in a virtual environment would not only allow its continued administration during pandemic restrictions but could also enable both therapy and research in spaces geographically separated from a centralized center of expertise. In this way, remote monitoring of NMES therapy could increase equity in health care delivery and research by removing location and travel costs as barriers to individual participation.

The primary objective of this pilot study was to assess the feasibility of administering a virtually guided NMES protocol of the tibialis anterior (TA) muscle while capturing HRV, quality of contraction (QoC), and NPRS data in healthy participants. Our secondary objectives were to investigate if HRV differs when NMES is administered ON and OFF the motor point of the TA muscle and to determine if there is a correlation between HRV with NPRS ratings during NMES. The findings from this study can be used to inform larger-scale studies that utilize HRV to monitor comfort in a non-responsive patient during NMES therapy and to lay the foundation for NMES therapy in patients who may not be able to attend centralized treatment centers for therapy or research purposes. 

## 2. Materials and Methods

This study was approved by Unity Health (Protocol # 20-337) and the University of Toronto (Protocol # 00041156) Research Ethics Boards in Toronto, Ontario, Canada, in accordance with the Declaration of Helsinki. All participants provided written informed consent prior to participating in the study.

### 2.1. Participant Recruitment

Participants were eligible for the study if they were a first- or second-year Physical Therapy student from the University of Toronto, 18 years of age or older, and received formal training in the application of NMES. Twelve (n = 12) healthy participants were recruited via digital posters and email. Participants were excluded from the study if they had a history of neuromuscular disease, lower extremity joint trauma in the preceding two years, and a history of fainting. Participants who had a pacemaker or implanted defibrillator, were pregnant, were living with an active malignancy, had a chronic disease impacting physical function, had a chronic skin condition for which topical steroid prescription medications were required (e.g., psoriasis, eczema), were requiring the use of an ambulatory aid, or were using systemic corticosteroids six months prior to the study were also excluded.

### 2.2. Virtual Data Collection Setup

**Figure 1:**
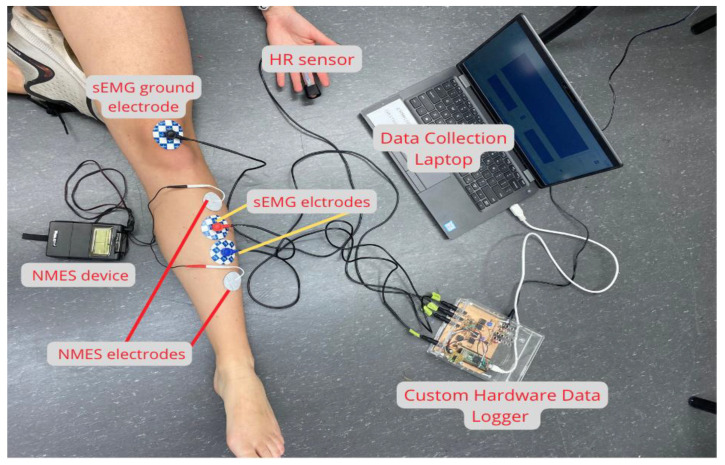
Participant data collection setup displaying the electrodes, NMES device, HR sensor, data logger, and laptop. Surface electromyography (sEMG) was collected; however, due to technical challenges with the EMG signal, EMG data are not reported in this study.

Study participants were provided with a sanitized data collection kit delivered to the participant’s location. The data collection kit contained sanitation supplies, a printed NPRS, a custom hardware data logger, hardware connector cables with labels, disposable batteries, a skin marker, a shaving razor, a tape measure, a laptop (Dell, Austin, TX, USA), 38 mm × 32 mm surface EMG electrodes (MedGel Pediatric Foam Electrodes, Medline, Northfield, IL, USA), a motor point pen (Compex, Mississauga, ON, Canada), NMES electrodes (Bodymed, Hudson, OH, USA), an NMES device (TwinStim Plus 2nd Edition Combo EMS digital electrotherapy unit, Medelco, Toronto, ON, Canada), and a photoplethysmography (PPG) heart rate sensor (World famous Electronics, Brooklyn, NY, USA) (Figure 1).

Virtual data collection was completed via video conference using the Zoom platform (Zoom Video Communications, Santa Clara, CA, USA). The custom hardware data logger was connected to the laptop. Labview 2018 (National Instruments Corporation, Austin, TX, USA) was used to acquire the data. The camera of the laptop was oriented to enable visual representation of the experimental protocol and data collection procedure by the remote supervising researcher (Figure 1), who counselled and monitored the participants through testing setup and the experimental procedure. Data were stored and encrypted on a secure server at the Department of Physical Therapy at the University of Toronto. 

### 2.3. Skin Preparation

Each participant verbally identified their dominant leg as the leg that they use to kick a ball. With the participant’s dominant leg exposed, the skin overlying the TA muscle was shaved and cleaned with alcohol. In order to reduce potential electrical impedance, participants were also asked not to apply any creams or moisturizers on their skin on the day of testing.

### 2.4. Anthropometric Data

Participants verbally reported their age, sex, height, and weight. Researchers guided the participants to measure their shank length (from the top of the fibular head to the lateral malleolus) and shank girth (around the thickest area of the calf). 

### 2.5. Motor Point Localization

Participants were guided to find the motor point of their TA using a motor point pen according to the protocol described by Gobbo et al., 2014 [9], and asked to mark the motor point with an “X” using a skin marker. Participants were guided to identify a location off the motor point of the TA muscle, defined as a point corresponding to one-third the shank length, proximal to or distal from the identified motor point.

### 2.6. Stimulation Electrodes

Using a randomization table (www.randomization.com (accessed 27 October 2021)), participants were randomly assigned to start the NMES in either the “ON” or “OFF” motor point of the muscle. Participants placed one electrode on the motor point of the muscle (“ON”) or off the motor point of the muscle (“OFF”) depending on their allocation. The second electrode was placed distally from the first electrode (Figure 1) in both ON and OFF electrode configurations. 

### 2.7. Heart Rate Data

Heart rate (HR) data were collected using a photoplethysmography (PPG) sensor placed on the participant’s right middle finger. HR data were sampled at 5000 Hz.

### 2.8. Quality of Contraction and Self-Reported Pain

The QoC of the TA muscle during the self-administered NMES and during voluntary activation was assessed remotely. The QoC was assigned a value of 0 (adequate contraction not elicited) or 1 (adequate contraction elicited). The criterion for an adequate contraction was defined as a smooth and visible contraction of the TA, in which the ankle moved into full available dorsiflexion range within more than half the available range with no accessory ankle movements (inversion, eversion, and plantarflexion). 

During each contraction, participants were asked to verbally self-report their degree of pain using an NPRS from 0–10 with anchor points of 0 = no pain and 10 = worst pain imaginable [17].

### 2.9. Baseline Trials

Participants were positioned in a long sitting position with a roll placed under the dominant leg (knee in slight flexion). Participants were asked to remain still for a period of 90 s while HR, QoC, and NPRS baseline measures were collected. 

Participants were asked to perform three successive voluntary contractions into full ankle dorsiflexion lasting 6 s each with 6 s of rest in between each contraction while HR data and QoC measures were collected.

### 2.10. Stimulation Trials

The NMES was delivered to the participant’s TA using a 50 Hz biphasic symmetric waveform with a pulse duration of 300 µs for a total of 6 s (2 s ramp-up time, 2 s hold time, 2 s ramp-down time). A 6 s rest period was provided between each stimulation cycle. The NMES intensity was gradually increased to elicit an adequate tetanic muscle contraction. This intensity was recorded and used for the duration of the participant’s trials in both the ON and OFF motor point stimulation trials. If a participant reported excessive discomfort prior to reaching a quality contraction, the maximum intensity they were able to tolerate was used for the stimulation trials. 

Each participant completed three 90 s trials of NMES ON and OFF the muscle motor point. Each trial consisted of 7–8 stimulation cycles (6 s on time, 6 s off time). During the peak intensity of the 6 s stimulation cycle, the QoC was recorded. During the rest period (6 s off time), the participant was asked to report their level of discomfort during the preceding stimulation period (i.e., on time) using the NPRS. HR data were continuously collected throughout the 90 s trial. In order to minimize muscle fatigue, a two-minute rest period was provided between each trial.

### 2.11. Kit Disconnection and Cleaning

Following data collection, the participant removed the surface and stimulation electrodes and disconnected the hardware connections. The participant was guided to repackage the data collection kit. The kit was then retrieved by the research team, sanitized, and restocked for use by the next participant.

### 2.12. Heart Rate Data Processing

HR data were processed in Matlab R2022a (MathWorks, Torrance, CA, USA). A low-pass filter of 10 Hz was used to isolate systolic peaks, which were then plotted and visually inspected by the researchers to confirm that all peaks within a given trial were appropriately detected. An HRV analysis was conducted by first calculating the beat-to-beat time interval [18]. The calculated beat-to-beat data interval signal was interpolated using the cubic spline interpolation function in Matlab and resampled at equal time intervals at 4 Hz. A power spectrum density analysis was conducted on the resampled data using Welch’s power spectrum density estimator function in Matlab. The area under the signal power versus frequency curve for the spectral bands for low frequency (LF) (0.04–0.15 Hz) and high frequency (HF) (0.15–0.40 Hz) was calculated. The ratio of LF/HF was calculated as outlined in Sesay et al. (2015) [11]. HRV data (LF/HF ratio) from each 90 s trial were expressed as a percentage of each participant’s baseline LF/HF ratio (%LF/HFbaseline).

### 2.13. Statistical Analysis

HRV, QoC, and NPRS data for three contractions from each 90 s trial were used in the analysis. SPSS Statistics version 28 (IBM, Armonk, NY, USA) was used for statistical analysis. Data normality was determined using the Shapiro–Wilk test. Means and standard deviations were calculated for continuous variables. Frequencies were calculated for categorical variables. The HRV (%LF/HFbaseline) and NPRS data from each trial were averaged for each participant. The mode of QoC data from stimulation trials was calculated for each participant. 

Differences in mean HRV and NPRS between both stimulations ON and OFF the motor point were assessed using a two-tailed paired *t*-test. The difference between the QoC in both conditions was assessed using the McNemar test. Pearson’s correlation was used to determine potential correlations between pooled (stimulation ON and OFF the motor point) HRV and NPRS data. Correlation coefficients were interpreted as follows: *r* = 0.21–0.40, poor correlation; *r* = 0.41–0.6, moderate correlation; *r* = 0.61–0.8, good correlation; and *r* > 0.81, very good correlation [19]. Significance was set at *p* < 0.05.

## 3. Results

### 3.1. Participants

Participant demographic and anthropometric data are shown in Table 1. Seven males and five females participated in this study. 

### 3.2. Quality of Contraction

**Figure 2:**
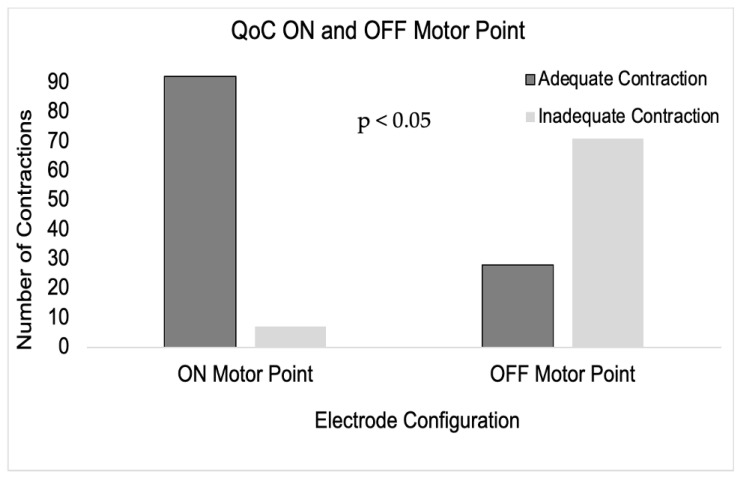
Frequency count of observed QoC (adequate or inadequate) during stimulation of TA muscle ON or OFF the motor point. The frequency of adequate contractions was significantly greater when the muscle was stimulated ON the motor point when compared with OFF the motor point (*p* < 0.05).

The number of adequate contractions during NMES ON and OFF the motor point is shown in Figure 2. The number of adequate contractions was significantly greater when the muscle was stimulated ON the motor point when compared with stimulation OFF the motor point (Figure 2). 

### 3.3. NPRS 

**Figure 3:**
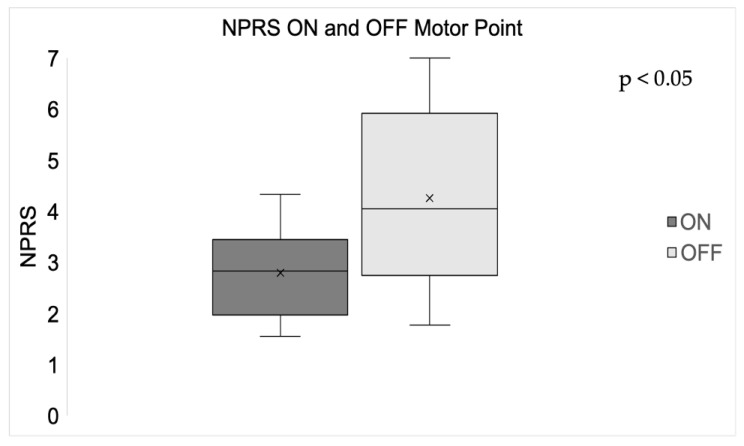
Boxplots of mean NPRS ratings when stimulating ON and OFF the motor point of the muscle. NPRS was significantly less when muscles were stimulated ON the motor point (*p* < 0.05).

The participant perception of pain was significantly less when the simulation was delivered ON the motor point when compared with stimulation OFF the motor point (Figure 3).

### 3.4. Heart Rate Variability

Noise from the HR signal from two participants could not be removed during the ON and OFF motor point configuration. Noise from the HR signal from an additional two participants could not be removed during the OFF motor point configuration. Consequently, systolic peaks in all the trials could not be identified accurately, resulting in missing data from two participants during the ON motor point configuration and four participants during the OFF motor point configuration. Thus, data from only eight participants were used in the analysis of HRV.

**Figure 4:**
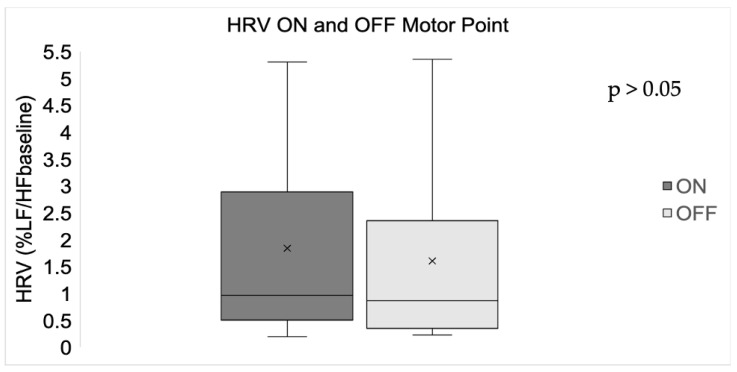
Boxplots of mean HRV (%LF/HFbaseline) when stimulating ON and OFF the motor point. No significant difference in HRV was found between stimulating ON and OFF the motor point (*p* > 0.05).

There was no significant difference between mean HRV (%LF/HFbaseline) when stimulating ON and OFF the motor point (Figure 4)

### 3.5. Correlation of HRV and NPRS

**Figure 5:**
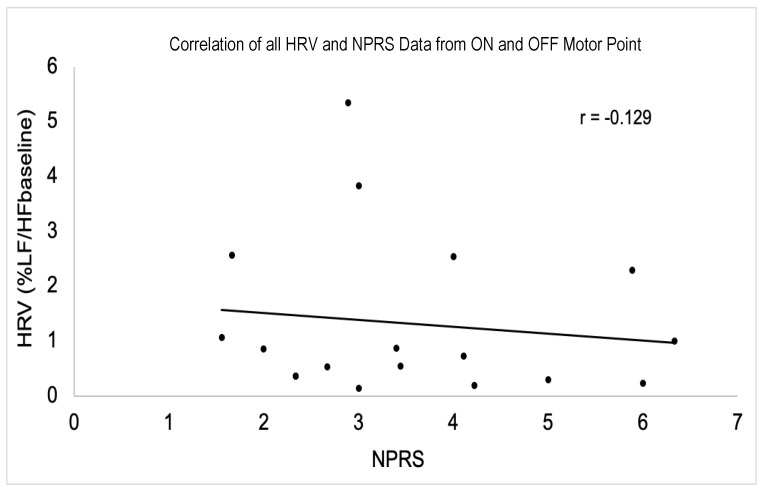
Absence of significant correlation between pooled participant HRV and NPRS data (*p* > 0.05). Participants 2 and 5 had identical data, resulting in one data point representing two participants on the graph.

There was no correlation between HRV and the NPRS (Figure 5).

## 4. Discussion

The purpose of this study was to assess the feasibility of administering a virtually guided NMES protocol of the TA muscle while capturing HRV, QoC, and NPRS data in healthy participants. In the present study, we were able to demonstrate that a cohort of healthy participants could be supervised virtually in real time and self-administer NMES to their TA muscle while capturing HRV, QoC, and NPRS data. Due to technical challenges, we were unable to capture EMG signals that were free from noise and could be processed and analyzed. The supervising scientist was able to view and easily grade the status of the muscle contraction remotely via video conference (Zoom) in all study participants.

We found an inadequacy of muscle contraction when the electrical stimulus was applied OFF the muscle motor point as opposed to ON the motor point of the TA muscle. The observation that the NMES generated a significantly greater number of adequate contractions when the TA muscle was stimulated ON vs. OFF the physiologic motor point was expected and consistent with previous work [9]. Electrical stimulus applied ON the motor point recruits a larger number of muscle motor units, thereby inducing greater muscle force, which translates into an adequate muscle contraction [9]. 

Our findings demonstrated greater patient comfort when the NMES was applied ON the motor point of the TA muscle vs. OFF the motor point of the TA muscle, with significantly lower scores in the NPRS, indicating less pain [17], consistent with the existing literature [9]. Stimulation of the motor point of the muscle has been shown to activate primarily the motor nerve [20]. Increased amplitude of the stimulation is needed when stimulating OFF the motor point to achieve the desired response [9]. Nociceptive receptors are in close proximity to the muscle motor unit and will be stimulated when NMES is not specifically targeted to the motor point, thereby eliciting greater discomfort or pain. 

We were able to remotely acquire the PPG signal for the vast majority of patients. However, systolic peaks of data from four participants could not be extracted due to a noisy signal. HRV was not significantly affected by the location of muscle stimulation, and we did not observe any correlations between HRV and pain perception. We had expected a higher %LF/HFbaseline when stimulating the muscle OFF vs. ON the motor point and correlation with the NPRS due to an anticipated increase in the sympathetic nervous system’s contribution to sinus rhythm, reflecting participant discomfort resulting from a noxious stimulus. 

HRV and derived metrics have been shown by others to serve as a proxy measure of the pain response elicited by mechanical, thermal, or post-surgical pain [11,21]. The present study, however, elicited pain using NMES. It is possible that the method of pain induction impacts the autonomic nervous systems response differentially, and this may account for the lack of an apparent impact on changes in HRV with stimulation ON and OFF the motor point and the absence of correlation between HRV and NPRS in our study. Alternatively, HRV metrics other than or combined with the frequency-domain variables analyzed here may serve as more sensitive pain proxy measures. For example, the Root Mean Square of Successive Differences (RMSSD) calculated from the beat-to-beat time interval has been shown to be a more reliable measure of HRV than frequency-domain measures, particularly for short-term measurements [19]. A combined RMSSD may be more suitable in exploring differences in HRV when stimulating the muscle ON or OFF the motor point. 

The findings from this work pave the way towards implementing NMES in non-responsive patients. Physiological metrics including HRV and the quality of muscle contraction may serve as important surrogate measures for appropriate NMES delivery in a non-responsive patient. We have established significant differences in the quality of muscle contraction when NMES is delivered ON vs. OFF the motor point of the TA muscle. This may be important in a non-responsive or ventilated patient who may not be able to report discomfort during NMES delivery, necessitating the use of the quality of contraction to determine adequate NMES application. Further work is needed to establish appropriate HRV metrics that relate to the pain response during NMES delivery.

### Limitations

Limitations are present within the current study. The QoC was virtually assessed using subjective criteria based on previous reports [10]. Although the researchers designated one member of the team to judge all contractions virtually to ensure consistency, and this was conducted with ease, variability may have occurred due to the subjectivity of appreciation of the extent of ankle movement. The incorporation of validated objective measures into the virtual environment, such as goniometric measurements of joint range of motion during the NMES, could reduce error and increase reliability. Although participants were guided verbally through the data collection protocol to identify the TA motor point, varied site identification is expected to exist due to inherent variability incumbent with different individuals completing the task. Importantly, all study participants were physiotherapy students with extensive knowledge of anatomy and formal training in administering NMES, which should have limited this variability. Additional guidance with motor point localization, such as a pictorial representation of possible motor point locations, would be required to streamline and better assist patients and members of the general population in motor point identification in a virtual environment. The generalizability of the study findings is also limited, as all participants were young, healthy, and physically active. HRV measures are known to differ between populations, e.g., sedentary vs. active individuals. The impact of this on the applicability of this study to an ICU population requires representation of diverse populations in the participant group. 

Given that the aim of the study was to execute NMES in a virtually guided environment, we were unable to confirm if participants adequately prepared their skin and placed sEMG electrodes in a secure manner over the TA muscle. Poor skin preparation and placement of the sEMG electrodes may have affected the sEMG signal quality and led to excessive noise. To improve EMG signal quality and the standardization of EMG data collection, direct observation of skin preparation by the researcher should be undertaken. Additionally, measurement of skin impedance, to ensure it is below 10 kOhm prior to data collection, should be considered [22].

## 5. Conclusions

This study demonstrates that NMES can be self-administered in a virtually guided environment while capturing metrics of comfort and muscle response, including HRV, QoC, and NPRS data, in healthy participants. We were able to validate the efficacy of NMES delivery and data acquisition in the virtually guided environment by reproducing study findings that report increased pain and inadequate quality of muscle contraction with NMES delivered OFF vs. ON the motor point. Future studies to further optimize the use of NMES in a remote capacity for both therapeutic and research endeavors have the potential to vastly increase equity in health care delivery and participation in research, as the geographic location of the patient relevant to health care expertise becomes irrelevant. While we did not find that the HRV LF/HF ratio adequately identified pain, further work in this regard is essential to identify physiologic variables that can be used in the absence of verbal communication to ensure that patients with depressed levels of consciousness are able to receive NMES therapy, such as a critically ill individual at risk for ICUAW. Additionally, the use of time-domain measures of HRV may be more appropriate than frequency-domain measures for capturing HRV changes during NMES trials. Furthermore, determining value labels of physiologic metrics that also enable tracking of the quality of muscle contraction could enable the use of algorithms and artificial intelligence to develop remote monitoring and automation tools for further efficiency of NMES delivery. Reducing the labor intensity of NMES delivery from a human resource standpoint would increase the possibility of its application in patient populations at risk for loss of muscle mass and strength. 

In order to ensure the success of a virtually guided NMES data collection protocol, hardware must be streamlined to ensure proper sensor attachment, and increased technical troubleshooting guidance should be given to researchers. However, a virtual protocol requiring advanced technological and anatomy knowledge would likely need to be simplified, and more guidance for participants would need to be provided in order to expand the protocol to the general public. Future virtual studies should be conducted to enhance the design and improve the feasibility of the virtual protocol before data collection with various patient populations and eventually in the ICU.

Ultimately, streamlining the protocol through improved hardware, the use of a more appropriate HRV metric, improved participant and researcher guidance, and the use of an objective QoC measure is recommended to increase the quality of virtual data collection in future studies.

## Figures and Tables

**Table 1:** Demographic and anthropometric data of study participants (n = 12).

Variables	Mean ± SD
Age (years)	24.2 ± 1.7
Weight (kg)	67.6 ± 14.4
Height (cm)	168.8 ± 9.1
Shank circumference (cm)	36.9 ± 3.5
Shank length (cm)	38.1 ± 3.1

## Data Availability

The datasets presented in this article are not readily available because the data are part of an ongoing study and cannot be shared due to ethical requirements. Requests to access the datasets should be directed to shar.gabison@utoronto.ca.
